# Adoptive cell therapies in solid tumors: current clinical landscape, challenges, and future directions

**DOI:** 10.3389/fimmu.2026.1800292

**Published:** 2026-05-14

**Authors:** Joe Rizkallah, Bahaa El Deen Wehbeh, Waseem Wassouf, Elio Salameh, Adnan Joumaa, Francois Jose Haddad, Lama Hamade, Nicole Charbel, Ghada M. Bakri, Ali Awada, Firas Kreidieh

**Affiliations:** 1 Department of Diagnostic Radiology, American University of Beirut, Beirut, Lebanon; 2 Division of Hematology and Oncology, Department of Internal Medicine, American University of Beirut, Beirut, Lebanon; 3 Division of Cardiology, Department of Medicine, Johns Hopkins University, Baltimore, MD, United States; 4 Faculty of Medicine, Saint-Joseph University of Beirut, Beirut, Lebanon

**Keywords:** adoptive cell therapy, cancer immunotherapy, CAR-T cells, immunoregulation, solid tumors, T cell receptor therapy, tumor microenvironment, tumor-infiltrating lymphocytes

## Abstract

Adoptive cell therapy (ACT) has emerged as a transformative strategy in cancer immunotherapy, offering durable clinical benefit in hematologic malignancies and expanding therapeutic potential in solid tumors. However, the translation of ACT to solid malignancies remains constrained by biological, immunological, and logistical challenges. This narrative review provides an evidence based overview of the current clinical landscape of ACT in solid tumors, with a focus on chimeric antigen receptor T cell (CAR-T), tumor-infiltrating lymphocyte (TIL), and T cell receptor–engineered T cell (TCR-T) therapies. We summarize recent clinical trial outcomes, highlight tumor-specific antigen targets, and examine key determinants of therapeutic efficacy across major solid tumor types. The review discusses central obstacles limiting ACT success in solid tumors, including antigen heterogeneity, immune evasion, inadequate T cell trafficking, limited persistence, and functional exhaustion within the immunosuppressive tumor microenvironment. Mechanisms driving treatment resistance, on-target off-tumor toxicity, and immune-related adverse events such as cytokine release syndrome and immune effector cell–associated neurotoxicity syndrome are critically evaluated. We further examine evolving strategies designed to overcome these barriers, including multi-antigen targeting, armored and logic-gated CAR designs, metabolic and cytokine engineering, locoregional delivery approaches, and next-generation manufacturing platforms incorporating allogeneic and gene-edited products. In parallel, the role of biomarkers, tumor microenvironment profiling, and personalized treatment selection is explored as a means to optimize patient stratification and enhance therapeutic outcomes. Advances in translational research, combination immunotherapy, and precision immuno-oncology are positioned as key drivers of the next phase of ACT development. By integrating mechanistic insights with emerging clinical evidence, this review outlines the progress, limitations, and future directions of ACT in solid tumors. It aims to provide a forward-looking framework to guide ongoing research, clinical trial design, and the rational implementation of adoptive cellular immunotherapies in solid malignancies.

## 1 Introduction

Solid tumors pose major challenges in oncology. This is partly attributable to intrinsic treatment resistance, antigen heterogeneity, and a complex immunosuppressive tumor microenvironment (TME), despite substantial advances in surgery, chemotherapy, radiotherapy, and checkpoint targeted immunotherapies (1). Adoptive cell therapies (ACT), including chimeric antigen receptor T cells (CAR-T), tumor-infiltrating lymphocytes (TIL), and T cell receptor–engineered T cells (TCR-T), are emerging as promising approaches for treating advanced solid tumors (2).

CAR-T therapies have revolutionized the treatment of hematologic malignancies; consequently, interest in applying ACT to solid tumors has expanded. However, antigen heterogeneity and the suppressive nature of the TME have limited the efficacy of CAR-T therapies in solid tumors. TIL therapy, by contrast, has demonstrated promising activity in advanced melanoma, particularly in combination with immune checkpoint inhibitors. Nonetheless, biological, logistical, and economic barriers have constrained its broader implementation. Similarly, TCR-T therapies are progressing rapidly, with multiple clinical trials targeting shared solid tumor antigens (3, 4).

Despite these encouraging developments, several limitations remain. In addition to adverse events commonly observed in hematologic malignancies, such as cytokine release syndrome (CRS) and immune effector cell–associated neurotoxicity syndrome (ICANS), which are generally manageable, on-target off-tumor (OTOT) toxicity represents a major safety concern in solid tumor settings (4). Furthermore, logistical barriers and the manufacturing complexity of patient-derived autologous therapies restrict widespread clinical adoption (1, 5).

In this narrative review, we aim to provide an evidence based overview of the current clinical landscape of ACT in solid tumors, focusing on CAR-T, TIL, and TCR-T therapies. We summarize key successes, ongoing trials, and emerging evidence of clinical benefit. Additionally, we discuss the distinct challenges faced by ACT in solid tumors, including the immunosuppressive TME, impaired trafficking, limited persistence, antigen selection constraints, and treatment related toxicities. Finally, we explore future strategies to address these barriers, such as next-generation ACT platforms, combination approaches, enhanced T cell persistence, novel delivery methods, and the development of predictive biomarkers.

This narrative review was conducted through a structured literature search of PubMed/MEDLINE and ClinicalTrials.gov, covering publications from January 2015 through June 2025, while including earlier landmark studies. The following search terms were included: “adoptive cell therapy,” “CAR-T,” “tumor-infiltrating lymphocytes,” “TCR-engineered T cells,” “solid tumors,” “tumor microenvironment,” “cytokine release syndrome,” “on-target off-tumor toxicity,” and “clinical trials”. Study selection was based on clinical relevance, trial phase (with preference for Phase I - III trials), and the degree of contribution to understanding the mechanism of actions of ACTs. However, given the narrative design of this review, the literature selection process carries a risk of selection bias, and is not comprehensive, hence conclusions should therefore be interpreted with caution.

## 2 Current clinical landscape of adoptive cell therapies in solid tumors

### 2.1 CAR-T cell therapy

CAR-T cell therapy is an adoptive cellular immunotherapy that uses T lymphocytes genetically engineered to express a synthetic receptor (6, 7). A CAR typically includes an extracellular antigen-recognition moiety, often a single-chain variable fragment (scFv), linked to intracellular signaling domains, notably CD3 (Signal 1) and costimulatory domains (Signal 2; e.g., CD28 or 4-1BB), enabling T cells to recognize and eliminate tumor cells independently of major histocompatibility complex (MHC) presentation (7, 8). This approach has achieved remarkable success in hematologic malignancies, leading to multiple FDA approvals targeting antigens such as CD19 and BCMA in lymphomas, B cell acute lymphoblastic leukemia, and multiple myeloma, respectively (6, 8, 9). Translating this success to solid tumors has been considerably more challenging because of anatomical barriers that limit T cell trafficking, the immunosuppressive TME, and antigenic heterogeneity (6, 8, 10, 11). Despite these obstacles, recent clinical trials have demonstrated feasibility and encouraging efficacy signals, highlighting the potential for expanding CAR-T into solid tumors (7, 9, 10).

Across most histologies in solid tumors, the majority of published early-phase CAR-T trials have reported objective response rates (ORRs) below 20%, indicating the significant impact of TME-mediated immune suppression and antigen heterogeneity (8, 10). However, exceptions have emerged in tumors with homogeneous, tumor-restricted antigen expression or those responsive to locoregional delivery. Those exceptions include gastric/gastroesophageal junction (GEJ) cancer, which achieved an ORR of 26% with claudin (CLDN) 18.2-targeted CAR-T (satri-cel) in a randomized Phase II trial (12); pediatric neuroblastoma, which achieved an ORR of 63% with GD2-CART01 (13); and recurrent glioblastoma, with 62% of patients showing tumor regression with bivalent intracerebroventricular EGFR/IL-13Rα2 CAR-T (10, 12–15). Moreover, in metastatic castration-resistant prostate cancer (mCRPC), armored CAR-T approaches such as PSMA-targeting T cells that were engineered with a dominant-negative TGF-β receptor (CART-PSMA-TGFβRDN), achieved a disease control rate of 38.5% and PSA reductions of at least 30% in 4 of 13 patients, including one patient with >98% PSA reduction (16). This study contributed to establishing TGF-β blockade as a viable armoring strategy for immunosuppressive solid tumor TMEs (16). Taken together, these data establish a clear pattern, that CAR-T success in solid tumors occurs when antigen selection is precise and its mode of delivery bypasses and overcomes systemic immunosuppressive barriers (8, 10, 16).

Effective CAR-T cell therapy initially relies on identifying tumor-associated antigens (TAAs) that are predominantly expressed by malignant cells to minimize severe OTOT, which is a major challenge in solid tumor treatment (6, 8). Key targets in epithelial tumors include mesothelin (MSLN), which is widely expressed in pancreatic ductal adenocarcinoma (PDAC), ovarian cancer (OC), and malignant pleural disease (8, 17–19). Although MSLN is overexpressed in more than 80% of these cancers, its low-level expression in normal tissues confers an inherent OTOT risk (11, 19). Another important target is claudin 18.2 (CLDN18.2), which has been investigated in gastric and gastroesophageal junction (GEJ) cancers, because of its stomach-specific expression in approximately 70% of gastric adenocarcinomas (12). CLDN18.2-targeted CAR-T therapy has demonstrated a strong clinical signal, particularly in patients with high antigen density (H-score ≥75 by IHC), where elevated expression correlates with improved ORR (12).

In contrast, targeting carcinoembryonic antigen (CEA) in colorectal and pancreatic cancers raises concerns because of physiological expression in normal gastrointestinal mucosa, which has led to complications such as severe colitis and diarrhea in clinical trials and has motivated development of hypoxia-responsive CARs to improve safety (8, 10).

In neuro-oncology, major CAR-T targets include GD2, EGFR/EGFRvIII, and IL-13Rα2 (14, 18, 20). GD2 is highly expressed in high-risk neuroblastoma, diffuse midline gliomas (DMG), particularly H3K27M-mutated tumors, and SCLC/NSCLC, with limited expression in normal tissues (18, 20). This tumor-restricted expression profile has made GD2 an attractive target, and several studies have demonstrated antitumor efficacy and tumor reduction in both pediatric and adult populations (10, 20).

EGFR and its mutant form EGFRvIII, along with IL-13Rα2, represent key targets in glioblastoma (GBM). EGFR is broadly overexpressed in GBM, NSCLC, and triple-negative breast cancer (TNBC), whereas EGFRvIII is a GBM-specific oncogenic variant (14, 21). IL-13Rα2 is present in up to 75% of GBM cases, making it a frequent therapeutic target (14). To address tumor heterogeneity, bivalent CAR constructs targeting EGFR/EGFRvIII and IL-13Rα2 have been developed (14, 22).However, because EGFR is expressed in normal epithelial tissues, these approaches raise safety concerns and pose OTOT risks (8, 21, 23).

Toxicity remains a major barrier to CAR-T use in solid tumors. A notable example is carbonic anhydrase IX (CAIX), a key target in clear cell renal cell carcinoma (ccRCC) (8). Although CAIX is highly expressed in tumor tissue (more than 90% of ccRCC cases), it is also present on bile duct epithelium, leading to dose-limiting hepatobiliary toxicity and premature termination of a phase I trial (8). Another example is HER2-directed CAR-T that uses a high-affinity scFv antigen and caused fatal pulmonary toxicity in the first patient, attributed to HER2 expression on normal lung epithelium (8). Hence reconstruction with lower-affinity antigens was needed to restore an acceptable safety profile (8). These lessons have shaped the current antigen selection criteria, focusing on quantitative comparative expression analysis between tumor and normal tissues prior to clinical advancement (8). Other relevant targets currently advancing in early-phase trials include PSMA (mCRPC), DLL3 (SCLC), MUC1 (NSCLC, HNSCC), and GPC3 (HCC) (7, 8, 18, 20). **Table 1** summarizes CAR-T targets investigated in solid tumors.

**Table 1:** Summary of CAR-T cell therapy targets investigated in solid tumors. .

Target	Tumor types	Rationale & specificity	Safety concerns (OTOT)	Key reference(s)
Mesothelin (MSLN)	OC, PDAC, NSCLC, TNBC	Overexpressed in >80% of epithelial OC and PDAC; limited normal expression	Low-level normal tissue expression; Grade 2 CRS noted	(8, 11, 17–19)
Claudin 18.2 (CLDN18.2)	Gastric/GEJ cancer	Stomach-specific isoform in ~70% of gastric adenocarcinomas; high antigen density selects responders	CRS 95% (Grade 1–2); manageable	(12)
GD2	Neuroblastoma, DMG, SCLC/NSCLC	Highly expressed in tumor cells; limited normal expression; antigen density threshold determines safety window	No OTOT in DMG trial despite normal brain GD2 expression	(20)
EGFR/EGFRvIII/IL-13Rα2	GBM, NSCLC, TNBC	EGFR overexpressed in GBM; EGFRvIII is GBM-specific; IL-13Rα2 in ≥75% of GBMs	Normal epithelial EGFR expression; neurotoxicity with locoregional delivery	(14, 15, 21)
HER2	Breast, Sarcoma, GBM, Gastric	Overexpressed in 15–20% of breast cancers and several solid tumors	Fatal pulmonary toxicity (high-affinity scFv); mitigated by affinity tuning	(8)
PSMA	mCRPC	Lineage-restricted prostate target; validated with TGFβ-insensitive armored CAR-T	38.5% DCR; 1 fatal grade 4 CRS; armoring strategy addresses TME immunosuppression	(16)
CAIX	ccRCC	Expressed in >90% of ccRCC; limited normal tissue	Grade 2–4 hepatobiliary toxicity; trial terminated early	(8)
CEA	CRC, NSCLC, PDAC	Overexpressed in CRC	Severe colitis/diarrhea due to normal GI mucosa expression	(8, 10)
CLDN6	Germ cell, ovarian, endometrial	Largely absent from normal adult tissues	Manageable safety; minimal OTOT; BNT211 trial	(24, 25)
DLL3	SCLC	Extensively expressed on SCLC cell surface	Limited preclinical off-target toxicity	(8, 20)
GPC3	HCC	Overexpressed in HCC; absent from normal cells	33% ORR with IL-15 armored GPC3 CAR-T; 66% DCR	(9)

CAR-T, chimeric antigen receptor T cell; OTOT, on-target off-tumor toxicity; OC, ovarian cancer; PDAC, pancreatic ductal adenocarcinoma; NSCLC, non-small cell lung cancer; TNBC, triple-negative breast cancer; GEJ, gastroesophageal junction; DMG, diffuse midline glioma; GBM, glioblastoma; mCRPC, metastatic castration-resistant prostate cancer; SCLC, small cell lung cancer; HCC, hepatocellular carcinoma; CRS, cytokine release syndrome; ccRCC, clear cell renal cell carcinoma; CRC, colorectal cancer; DCR, disease control rate.

The efficacy of CAR-T in solid tumors has primarily been evaluated in early-phase (phase I/II) clinical trials, focusing on safety, delivery optimization, and preliminary antitumor activity. Among these, a randomized controlled trial (RCT) evaluated satri-cel (CLDN18.2-specific CAR-T) in advanced gastric or gastroesophageal junction cancer (12). This phase II study enrolled 156 heavily pretreated patients and demonstrated significantly improved progression-free survival (3.25 vs. 1.77 months; HR 0.37, p < 0.0001) and objective response rate (26% vs. 4%) compared with treatment of physician’s choice (12). Toxicity was manageable, with CRS occurring in 95% of patients, all grade 1–2, and higher-grade adverse events largely limited to hematologic toxicities, primarily lymphopenia (12).In CNS malignancies, promising results have emerged from trials using locoregional delivery to overcome the blood–brain barrier (14, 21). A phase I trial evaluated bivalent CAR-T targeting EGFR epitope 806 and IL-13Rα2 in 18 patients with recurrent glioblastoma (rGBM), establishing a maximum tolerated dose of 2.5 × 10^7^ cells (15). Although acute grade 3 neurotoxicity occurred in 56% of patients, 62% of evaluable cases demonstrated tumor regression, including one partial response and one durable stable disease lasting more than 16 months (15). Earlier studies using intravenously administered EGFRvIII-directed CAR-T showed limited efficacy and antigen escape (23), highlighting the importance of intratumoral delivery.

In pediatric neuroblastoma, GD2-targeted CAR-T therapy (GD2-CART01) has also shown promising results (13). In a phase I–II trial including 27 children with high-risk disease, GD2-specific CAR-T achieved an objective response rate of 63%, with toxicities that were predominantly mild and reversible (6, 10, 13). CAR-T persistence was observed for at least three months in most evaluable patients, supporting durable engraftment and feasibility in this population (6, 10, 13).

In advanced PDAC, a phase I trial of anti-mesothelin CAR-T demonstrated that intravenous, intraperitoneal, and intrahepatic delivery was safe but produced limited efficacy (18). Mechanistic analyses identified CAR-T exhaustion mediated by ID3 and SOX4 as a contributor to treatment failure, while dual knockout of these factors enhanced persistence and antitumor activity in preclinical models (18). Another interesting approach is the combination of CLDN6-targeted CAR-T with an amplifying mRNA vaccine (BNT211) which demonstrated meaningful efficacy and carried a manageable safety profile across multiple refractory solid tumor histologies (24). This approach contributed to establishing mRNA-based co-stimulation as a viable strategy. **Table 2** summarizes relevant clinical trials of CAR-T in solid tumors.

**Table 2:** Summary of select clinical trials of CAR-T cell therapy in solid tumors. .

Antigen (product)	Tumor type	Phase	N	Key outcomes	Toxicity	Clinical significance
CLDN18.2 (satri-cel) (12)	Gastric/GEJ Cancer	Phase 2 RCT	156	mPFS 3.25 vs 1.77 mo (HR 0.37, p<0.0001); ORR 26% vs 4%; DCR 72%	CRS 95% (Grade 1–2); lymphopenia 98%	First positive RCT for CAR-T in solid tumors
GD2 (GD2-CART01) (13)	Neuroblastoma	Phase 1–2	27	ORR 63%; CAR-T persistence ≥3 months in most evaluable patients	Predominantly mild, reversible	Efficacy comparable to hematologic CAR-T; supports pediatric solid tumor feasibility
EGFR+IL-13Rα2 (bivalent) (14, 15)	Recurrent GBM	Phase 1	18	62% tumor regression (8/13 evaluable); 1 durable SD >16 mo; mPFS 1.9 mo; MTD 2.5×10^7^ cells	Neurotoxicity 56% Grade 3 (acute, 12–48h); no Grade 4/5	Locoregional ICV delivery feasible and bioactive; establishes MTD
Mesothelin (iCasM28z) + pembrolizumab (26)	Malignant Pleural Mesothelioma	Phase 1	27 (18 + pembro)	mOS 23.9 months; 1-year OS 83%; SD ≥6 months in 8/16 patients; 2 complete metabolic responses on PET	No high-grade CRS or ICANS; well tolerated intrapleurally	First proof-of-concept for intrapleural CAR-T + checkpoint blockade; converts cold tumors to responsive
PSMA (CART-PSMA-TGFβRDN) (16)	mCRPC	Phase 1	13 treated	DCR 38.5%; 4 patients with ≥30% PSA reduction; 1 with >98% PSA reduction (died grade 4 CRS/sepsis)	5/13 grade ≥2 CRS; 1 fatal CRS; lymphodepletion markedly enhanced expansion	First clinical validation of TGFβ-blocking armored CAR-T in a solid tumor
MSLN (A2B694, Tmod™) (27, 28)	Solid tumors (NSCLC, PDAC)	Phase 1/2	Ongoing	No DLT, no CRS/ICANS; CAR-T expansion and tumor infiltration confirmed; 1 CR in refractory NSCLC	No grade ≥3 toxicity to date	First clinical proof-of-concept for logic-gated AND-NOT CAR-T in solid tumors
CLDN6 (BNT211) (24)	Multiple solid tumors	Phase 1/2	Ongoing	Meaningful efficacy in refractory TGCT, ovarian, endometrial; response augmented by mRNA vaccine	Manageable; no dose-limiting OTOT	Validates tumor-selective target + mRNA vaccine strategy for CAR-T amplification

ICV, intracerebroventricular; mOS, median overall survival; mPFS, median progression-free survival; ORR, objective response rate; DCR, disease control rate; MTD, maximum tolerated dose; SD, stable disease; CR, complete response; DLT, dose-limiting toxicity; CRS, cytokine release syndrome; ICANS, immune effector cell-associated neurotoxicity syndrome; RCT, randomized controlled trial; PSA, prostate-specific antigen.

Given these encouraging clinical findings, the most notable advances in CAR-T have occurred in tumors with homogeneous antigen expression or in tumors amenable to locoregional delivery (8, 12). Gastric and gastroesophageal junction cancers, neuroblastoma, and glioblastoma have demonstrated the strongest clinical promise, marking a shift toward meaningful efficacy in solid tumors (8, 12). These successes highlight the importance of precise antigen selection (e.g., CLDN18.2, GD2) and locoregional administration strategies that enhance tumor accessibility while mitigating systemic toxicity (8, 12). Collectively, these findings suggest that the principles underlying CAR-T success in hematologic malignancies, particularly target specificity and persistence, can be adapted to the solid tumor context (8).

CAR-T is steadily evolving from an experimental strategy to a therapeutic reality in selected solid tumors. However, broader clinical implementation remains constrained by three major challenges: poor persistence and functional exhaustion within the immunosuppressive TME; OTOT arising from shared antigen expression with normal tissues; and inefficient trafficking and infiltration into dense stromal regions (8–10). Recent CAR-T trials in solid tumors suggest that the TME presents different challenges seen in hematologic malignancies, requiring new solutions rather than simple extrapolation of prior success. Hence, where significant response have emerged (i.e. neuroblastoma, GBM, gastric cancer, and malignant pleural mesothelioma) success can be attributed to precise antigen selection exploiting tumor-restricted or high-density expression, locoregional delivery bypassing systemic suppression, or engineered resistance to TME-specific immunosuppressive mechanisms such as TGF-β signaling (16). Future research should focus on enhancing CAR-T design and persistence, refining multi-antigen targeting to counter heterogeneity, and optimizing delivery approaches, including locoregional and combination therapies (7, 8, 11). These advances will be critical to realizing the full therapeutic potential of CAR-T in solid malignancies.

### 2.2 TIL therapy

TIL therapy represents the most clinically mature ACT modality in solid tumors, with TIL having a Level I evidence of superiority over systemic immunotherapy in melanoma (29). In the pivotal phase III C-144–01 trial, TIL therapy showed a median progression-free survival (PFS) of 7.2 months versus 3.1 months for ipilimumab (HR 0.50, p = 0.002), an ORR of 49% versus 21%, and a median overall survival of more than 25 months (29). This data contributed to the FDA approval of the first commercial TIL product, lifileucel (Amtagvi), which achieved an ORR of 31.5% with 43.5% of responders maintaining durable responses at 12 months (30). In refractory cervical cancer, TIL has demonstrated ORRs up to 28%, and an ORR of 18% in HPV-associated head and neck squamous cell carcinoma, with promising signs in NSCLC and endometrial cancer (31, 32). The rate of responses in tumors with no common targetable surface antigen reflects TIL’s core advantage, its polyclonal TCR repertoire, which enables simultaneous recognition of multiple tumor-specific neoantigens, and includes patient-specific mutations that antigen-directed CAR-T cannot target (31, 32).

TIL therapy is a form of ACT that uses autologous T cells isolated directly from a patient’s tumor tissue (33, 34). The process begins with surgical resection of a tumor specimen, after which lymphocytes that have naturally infiltrated the tumor microenvironment are harvested (33, 35). Subsequently, high-dose interleukin-2 (IL-2), reaching 6000 IU/ml, and in some protocols additional T cell receptor (TCR) stimulation and feeder cells, are used to expand TILs ex vivo, generating a large population of tumor-reactive T cells (33, 35–37). Following a lymphodepleting chemotherapy regimen, the expanded TILs are reinfused into the patient’s bloodstream, often in combination with systemic high-dose IL-2 to support *in vivo* persistence and activity (33, 38). This approach differs from other adoptive T cell therapies in that lymphocytes are derived exclusively from tumor tissue, unlike CAR-T and TCR-T therapies, which use genetically engineered peripheral blood T cells expressing synthetic receptors that recognize predefined cell-surface antigens or peptide–HLA complexes, respectively (6, 33, 39). Therefore, whereas CAR-T and TCR-T products are monoclonal or oligoclonal, TIL products are polyclonal and retain a diverse repertoire of native TCRs, enabling recognition of a broad range of tumor-associated antigens, including patient-specific neoantigens (31, 32).

The clinical role of TIL therapy is most firmly established in metastatic melanoma, where it has demonstrated durable and superior efficacy. Early work by Rosenberg et al. (40–42) at the National Cancer Institute (NCI) reported objective response rates (ORRs) of approximately 30–50% and long-term overall survival exceeding 20 months in responding, including heavily pretreated, populations. Subsequently, Seitter et al. (43) reported that among 48 patients with complete tumor regression following TIL therapy, 46 required no further treatment and achieved a 96% 10-year melanoma-specific survival. More recently, a multicenter phase III trial comparing TIL therapy with ipilimumab demonstrated a median progression-free survival of 7.2 months for TIL therapy versus 3.1 months for ipilimumab, with an ORR of 49% versus 21%, and a median overall survival exceeding 2 years in the TIL group (29). Lifileucel, the first FDA-approved TIL product for unresectable or metastatic melanoma, achieved an ORR of 31.5%, with 43.5% of responders maintaining durable responses at 12 months (30). In addition, multiple meta-analyses indicate that prior anti-PD-L1 therapy does not reduce TIL efficacy, supporting use in refractory disease (35, 44–46).

Beyond melanoma, TIL therapy is under active investigation in other solid tumors. Cervical cancer trials have reported ORRs up to 28% in refractory disease, with complete remissions persisting beyond five years after a single infusion (47). Based on these results, the FDA granted Breakthrough Therapy Designation for TIL therapy in cervical cancer. Early-phase trials in anti-PD-1–resistant NSCLC have also demonstrated objective responses, including durable complete responses, although efficacy appears lower than in melanoma (6, 48). HPV-associated head and neck cancers have shown responses in approximately 18% of treated patients, with occasional long-term remission (48). Triple-negative breast cancer is an emerging indication, with the most promising outcomes reported in HER2-positive tumors, particularly after chemotherapy (49). Although responses have been observed in ovarian and colorectal cancers, overall clinical benefit remains more limited, potentially reflecting challenges in TIL persistence and expansion (6, 48, 50).

Clinical eligibility for TIL therapy includes both general and tumor-specific criteria. Patients typically must have a histologically confirmed unresectable or metastatic solid tumor, at least one measurable lesion according to RECIST, and a surgically accessible tumor site suitable for TIL harvest (29, 51, 52). Adequate performance status, commonly ECOG 0–1 or WHO 0–1, is required to tolerate surgery and lymphodepleting chemotherapy (53, 54). Patients must also have sufficient cardiac, hepatic, renal, and hematologic function and no rapidly progressive comorbidities that would preclude high-dose IL-2 therapy (29, 51). General exclusion criteria include uncontrolled infections, autoimmune disease requiring immunosuppression, untreated CNS metastases, or poor functional reserve.

Tumor-specific selection criteria further refine eligibility. Phase III melanoma trials have included patients aged 18–75 years with stage IIIC/IV disease, at least one prior systemic therapy, and at least one resectable lesion for TIL harvest (29, 51). In breast cancer, TIL therapy appears most promising in triple-negative subtypes, particularly following neoadjuvant chemotherapy, where higher TIL reactivity and proliferative capacity have been reported (55, 56). Biomarkers and pathologic assessments also contribute to patient selection. High intratumoral TIL density, particularly CD8+ and Th1-polarized T cells, correlates with improved outcomes and is frequently used as a selection marker (57–59). In NSCLC, tumors enriched with B cell infiltration or immunosuppressive microenvironments may yield less functional TIL products (60). Subpopulation profiling (e.g., CD4+, CD8+, Treg) and functional assays assessing cytokine production and tumor reactivity are increasingly used to evaluate harvested TIL quality (52, 57, 58). However, no universally accepted standard for TIL quantification or functional assessment currently exists, and substantial heterogeneity persists in product quality and clinical response (61). Ongoing efforts aim to standardize TIL harvesting, expansion, and selection protocols across tumor types and to refine biomarker-driven stratification strategies.

### 2.3 TCR-engineered T cell therapy

By targeting intracellular tumor proteins presented on MHC molecules, TCR-T therapy accesses antigens that CAR-T cannot target (62–64). Current RCT data across several tumor types reflect this potential, as NY-ESO-1-directed TCR-T produced ORRs of 61% in synovial sarcoma (11/18 patients) and 55% in melanoma (11/20 patients), with durable complete responses in both (65, 66); afamitresgene autoleucel (afami-cel) which targets MAGE-A4 via HLA-A*02, achieved an ORR of 37% in SPEARHEAD-1 (19/52; 95% CI 24–51) including 39% in synovial sarcoma (67); HPV-16 E7–specific TCR-T achieved a 50% ORR in anti-PD-1 refractory HPV-associated malignancies (6/12 patients) (68); and a patient with KRAS G12D mutated metastatic pancreatic cancer achieved a 72% partial response by RECIST 1.1 following a single neoantigen-specific TCR-T infusion (69). This data suggests that TCR-T appears to be best suited for tumors with high cancer-testis antigen expression, viral oncoproteins, or actionable patient-specific neoantigens (65, 67–69).

TCR-engineered T cell therapy (TCR-T) is an adoptive cell therapy in which a patient’s T cells are genetically modified to express a defined, tumor-reactive αβ T cell receptor (TCR) (62). After leukapheresis, T cells are activated ex vivo and transduced, commonly using retroviral or lentiviral vectors, to introduce the transgenic TCR, then expanded and reinfused (63). Unlike CAR-T cells, TCR-T cells recognize intracellular tumor proteins as short peptides presented on major histocompatibility complex (MHC)/human leukocyte antigen (HLA) molecules, making this approach inherently HLA-restricted (64). This MHC-dependent recognition expands the target space beyond surface antigens to include cancer-testis antigens (e.g., NY-ESO-1, MAGE family), differentiation antigens (e.g., MART 1, tyrosinase), viral antigens (e.g., HPV E7), and mutation-derived neoantigens (65). Clinical protocols frequently combine TCR-T infusion with lymphodepleting chemotherapy to enhance engraftment and, in some cases, with high-dose IL-2 to support early expansion (65). Because introduced α and β chains can mispair with endogenous TCR chains and engineered receptors can exhibit high avidity, extensive receptor design and specificity testing are central to manufacturing and safety (64). A major practical limitation is that efficacy depends on intact tumor antigen processing and presentation, as well as the presence of the appropriate restricting HLA allele in the patient (64). Preclinical and clinical experience further indicates that antigen selection must prioritize tumor-selective expression, because T cells do not inherently distinguish malignant from healthy tissues when the target peptide-HLA complex is present in both (70).

NY-ESO-1 is one of the most clinically validated TCR-T targets in solid tumors, particularly in synovial sarcoma and melanoma (65). In a landmark NY-ESO-1 TCR-T trial (lymphodepletion plus high-dose IL-2 at 720,000 IU/kg to tolerance), objective responses occurred in 4 of 6 patients with synovial sarcoma and 5 of 11 patients with melanoma, including 2 complete responses in melanoma persisting beyond 1 year and a partial response in synovial sarcoma lasting 18 months (65). Longer follow-up from the same program confirmed durable activity, with objective responses in 11 of 18 (61%) NY-ESO-1–positive synovial sarcoma cases and 11 of 20 (55%) NY-ESO-1–positive melanoma cases (66). That follow-up also reported landmark survival estimates, with 3- and 5-year overall survival rates of 38% and 14% in synovial sarcoma and 33% and 33% in melanoma, respectively (66).

Building on cancer-testis antigen targeting, MAGE-A4–directed TCR-T has advanced into later-stage clinical testing in sarcomas using afamitresgene autoleucel (afami-cel) for HLA-A*02 patients with MAGE-A4–expressing tumors (67). In the SPEARHEAD-1 study (open-label, non-randomized phase II), afami-cel achieved an ORR of 37% (19/52; 95% CI 24–51) overall, including 39% (17/44; 95% CI 24–55) in synovial sarcoma and 25% (2/8; 95% CI 3–65) in myxoid/round cell liposarcoma (67). Patients were heavily pretreated (median of 3 prior lines), and the median follow-up was 32.6 months (IQR 29.4–36.1), supporting assessment of response durability beyond early imaging time points (67).

Beyond cancer-testis antigens, viral oncoproteins represent attractive shared targets. A first-in-human phase I trial of HPV-16 E7–specific TCR-T cells demonstrated objective responses in metastatic HPV-associated epithelial cancers (68). In that trial, dose escalation reached 1 × 10¹¹ engineered T cells without dose-limiting toxicity, and objective responses occurred in 6 of 12 patients (50%), including 4 of 8 with anti–PD-1–refractory disease (68). Neoantigen-specific TCR gene therapy offers a complementary precision strategy, exemplified by KRAS G12D targeting in metastatic pancreatic cancer (69). A single infusion of 16.2 × 10^9^ TCR-engineered T cells (HLA-C*08:02-restricted) produced a 72% partial response (RECIST 1.1) that remained ongoing at 6 months, with engineered cells persisting at more than 2% of circulating T cells at that time point (69). Recent emerging platforms now utilize AI-driven algorithms to predict high-affinity TCR sequences against patient-specific neoantigens (71, 72).

**Figure 1:**
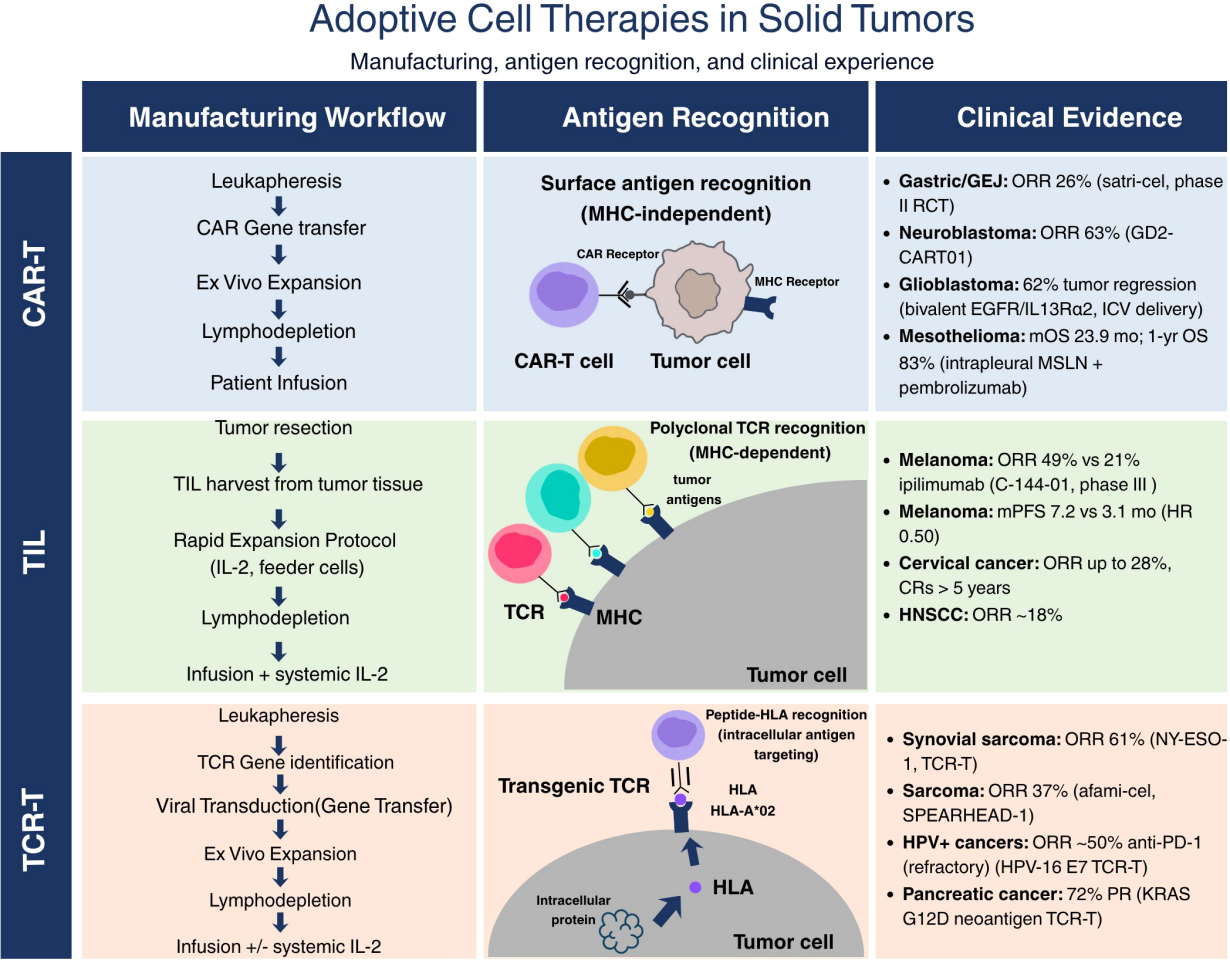
Overview of adoptive cell therapy modalities and clinical landscape in solid tumors. The three principal ACT platforms: CAR-T, TIL, and TCR-T are shown with their manufacturing workflows (left), mechanism of antigen recognition (center), and representative solid tumor indications with current clinical evidence benchmarks (right). CAR-T cells recognize surface antigens independently of MHC via a synthetic receptor; TIL products are polyclonal autologous T cells harvested from tumor tissue; TCR-T cells are engineered to recognize intracellular peptide–HLA complexes. Key response rates from pivotal trials are indicated for each platform. FDA, U.S. Food and Drug Administration; GEJ, gastroesophageal junction; GBM, glioblastoma; HLA, human leukocyte antigen; MHC, major histocompatibility complex; ORR, objective response rate; PFS, progression-free survival. References used (6, 7, 13–15, 26, 29, 30, 33, 35, 62, 63, 65–69).

Clinical development has also been shaped by critical safety lessons associated with engineered high-affinity receptors. In one trial, fatal cardiotoxicity occurred when the first two treated patients developed cardiogenic shock and died within days due to off-target recognition of a titin-derived peptide (73). Raman et al. later showed that structural molecular mimicry between MAGE-A3 and a titin-derived peptide underpinned this fatal off-target cardiotoxicity, emphasizing the difficulty of predicting clinically relevant TCR cross-reactivity (74). Similarly, in an anti–MAGE-A3 TCR trial, 9 patients were treated and 5 experienced tumor regression, but 3 developed acute mental status changes and 2 progressed to coma and died, highlighting the importance of stringent antigen selection and cross-reactivity screening (75). Current clinical and translational reviews therefore emphasize engineering strategies to improve efficacy in solid tumors while addressing HLA restriction, functional avidity, and TME barriers. **Figure 1** shows an overview of ACT modalities and clinical landscape in solid tumors.

## 3 Clinical challenges unique to solid tumors

### 3.1 Impaired trafficking, tumor penetration, and microenvironmental barriers

Although ACT has demonstrated promising potential in the treatment of hematologic malignancies, it continues to face substantial challenges in solid tumors. This difference is largely attributable to the ability of adoptive T cells (ATCs) to efficiently access malignant cells in blood-based cancers, resulting in higher treatment success and prolonged disease-free survival (76). In solid tumors, however, ATCs encounter multiple barriers after entering the circulation and during infiltration into the TME. These barriers include limited penetration of the tumor stroma, restricted accumulation within the tumor core, the immunosuppressive nature of the TME, and progressive exhaustion or dysfunction of ATCs (77).

ATCs cross blood vessels into the tumor core through extravasation mediated by selectins and integrins. However, tumor cells secrete angiogenic factors that induce structural abnormalities in the vascular endothelium (78). As a result, endothelial cells exhibit downregulated expression of adhesion molecules such as VCAM-1 and ICAM-1/ICAM-2 (79, 80). This process enables tumor cells to evade immune surveillance by restricting immune cell extravasation (79, 80). Immune cells that successfully extravasate must then overcome another defensive barrier: the dense tumor stroma (79, 80). Tumor cells secrete an interwoven network of collagen fibers within the extracellular matrix (ECM), which restricts immune cell motility and limits access to tumor antigens (81). In addition, cancer-associated fibroblasts (CAFs) produce metalloproteinases (MMPs) that remodel the ECM (82). These MMPs compartmentalize tumors into immune exclusion zones (83), causing ATCs to accumulate at tumor borders rather than infiltrating the tumor core (83). Furthermore, collagen rearrangement promotes ECM protein accumulation, increasing matrix density and organization (84). This stiffened and less permeable ECM creates a physical barrier that further impedes immune cell penetration and target engagement (84).

Multiple immunosuppressive cell populations, including regulatory T cells (Tregs), myeloid-derived suppressor cells (MDSCs), and tumor-associated macrophages (TAMs), populate the TME and play a central role in limiting ATC efficacy (85–88). These cells suppress antitumor immune responses through secretion of cytokines such as interleukin-10 (IL-10) and transforming growth factor beta (TGF-β) (85). Tregs accumulate at inflammatory and tumor sites and exert immunosuppressive effects through several mechanisms (86). They release TGF-β and IL-10, which inhibit T cell activity, including infused ATCs (87). Tregs also consume IL-2, a key immune growth factor, thereby limiting immune cell expansion and reducing the likelihood that ATCs reach malignant targets (85, 88). MDSCs further promote tumor progression and metastasis by suppressing immune activation and cytotoxicity (89). They exert these effects through secretion of nitric oxide synthase (iNOS), arginase-1 (Arg-1), IL-10, and IFN-γ (90). Nitric oxide produced via iNOS suppresses T cell proliferation and induces TCR nitration, impairing ATC responsiveness to tumor antigens (91, 92). Elevated Arg-1 expression depletes L-arginine within the TME, arresting the T cell cycle and limiting effector function (93). In addition, IL-10 and IFN-γ promote recruitment and expansion of Tregs and TAMs, reinforcing immunosuppressive signaling networks and tumor progression (94).

**Figure 2:**
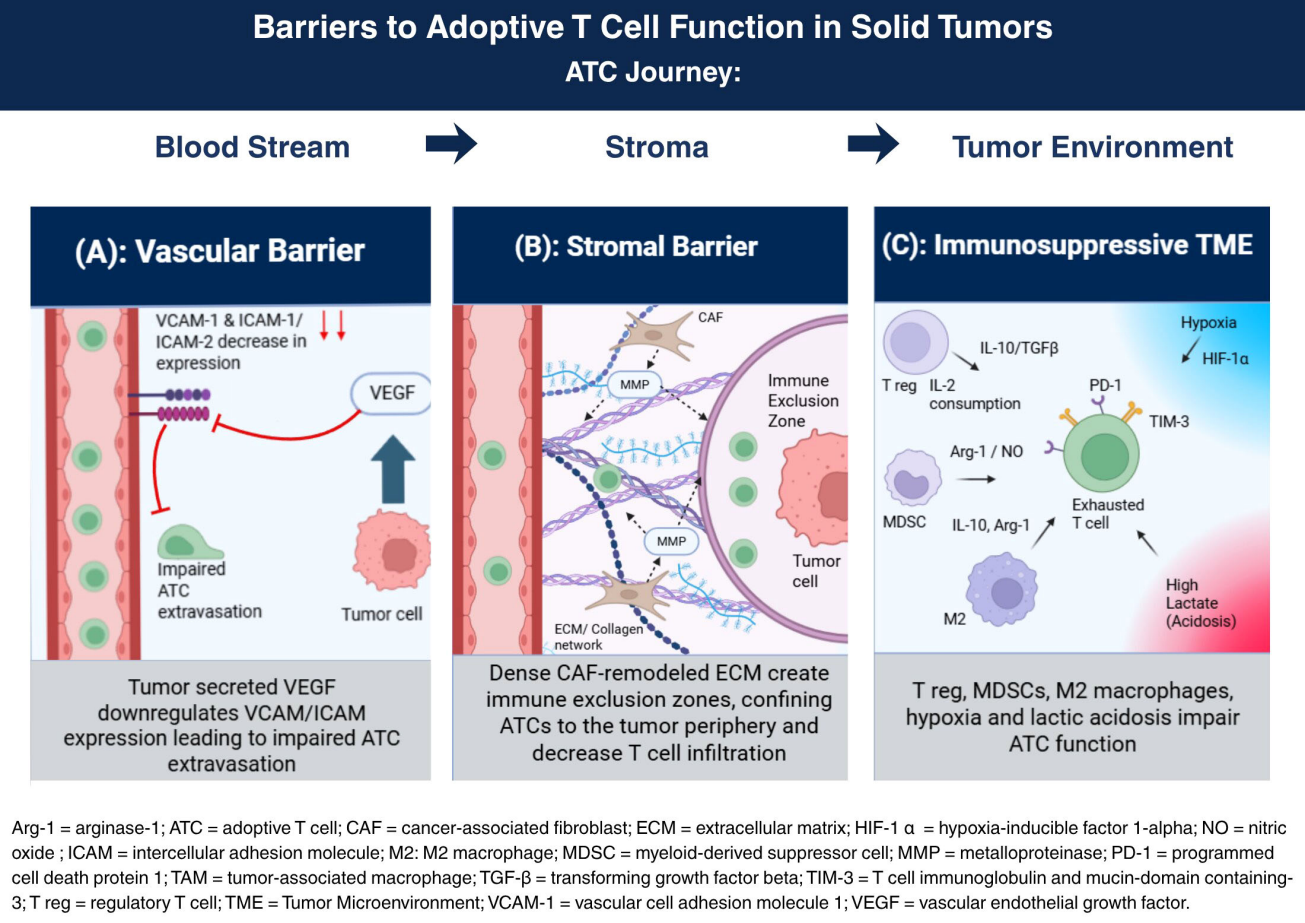
Barriers to adoptive T cell efficacy in the solid tumors. **(A)** Vascular barrier: tumor-secreted angiogenic factors downregulate endothelial VCAM-1 and ICAM-1/ICAM-2, restricting ATC extravasation. **(B)** Stromal barrier: CAF-remodeled ECM and collagen networks create immune exclusion zones, confining ATCs to the tumor periphery. **(C)** Immunosuppressive TME: Tregs, MDSCs, and M2-polarized TAMs suppress ATC function through TGF-β, IL-10, Arg-1, and nitric oxide secretion; hypoxia and lactic acid further impair ATC metabolic fitness (78–102).

TAMs, particularly M2-polarized macrophages, also contribute to immune suppression and tumor progression (95). Their production of IL-2 and Arg-1 suppresses immune cell activity and induces cell cycle arrest, respectively (96). TAMs additionally secrete MMPs that degrade and restructure the ECM, facilitating tumor cell migration while further restricting immune cell access and function (97). In contrast, M1-polarized TAMs exhibit proinflammatory activity that can enhance immunotherapy efficacy and prolong survival (98). Accordingly, therapeutic strategies aimed at improving ATC performance should seek to suppress M2 TAM activity while promoting M1 polarization. Beyond cellular immunosuppression, metabolic features of the TME further compromise ATC function. Rapid tumor growth increases oxygen demand, generating hypoxic conditions (99). Hypoxia impairs ATC oxidative phosphorylation and sustains hypoxia-inducible factors (HIFs), including HIF-1α, which suppress T cell glycolysis and reduce effector capacity (100). Hypoxia also limits T cell migration from blood vessels into tumor tissue, thereby decreasing infiltration efficiency (101). **Figure 2** summarizes the barriers to ACT efficacy in solid TME.

### 3.2 T cell exhaustion and antigen heterogeneity

Persistent antigen exposure within tumors drives T cells toward a dysfunctional, exhausted state characterized by reduced proliferation and impaired effector function (103). Because ATCs must remain active within the TME to eliminate malignant cells, exhaustion markedly diminishes therapeutic efficacy (104). Exhausted T cells exhibit reduced cytokine production and increased expression of inhibitory immune checkpoint receptors (105). For example, thymocyte selection–associated high mobility group box (TOX) becomes highly expressed following sustained TCR signaling (106), promoting upregulation of PD-1 and attenuating immune responses (107, 108). Persistent PD-1 expression is associated with functional T cell impairment and suppression of cyclin-dependent kinases (CDKs), along with downregulation of casein kinase 2 (CK2), which disrupts TCR signaling and inhibits cell cycle progression (109, 110). Additionally, elevated TGF-β within the TME suppresses T cell activation and proliferation by inducing SMAD signaling and PD-1 expression (111).

Metabolic byproducts produced by tumor cells further limit ATC persistence. Tumor cells rely heavily on anaerobic glycolysis, generating lactic acid as a metabolic byproduct (99). Accumulation of lactic acid acidifies the ECM, reducing T cell motility, survival, and immune synapse formation with tumor cells. Consequently, elevated lactic acid levels constitute a major barrier to effective immunotherapy by impairing ATC persistence and functional capacity (102). Importantly, recent findings in TOX-driven chromatin remodeling and NR4A family transcription factors offer new engineering opportunities to improve the fate of CAR-T cells after infusion (72, 112).

In contrast to leukemias, solid tumors present a major challenge in identifying tumor-specific markers that are truly homogeneously expressed. Through somatic mutation, tumor cells frequently give rise to multiple lineages with heterogeneous antigen expression (4). This indicates that even when a tumor-specific marker is initially identified, antigen-negative subclones or mosaic populations may emerge through clonal evolution, limiting the long-term efficacy of marker-specific CAR-T therapy.

**Figure 3:**
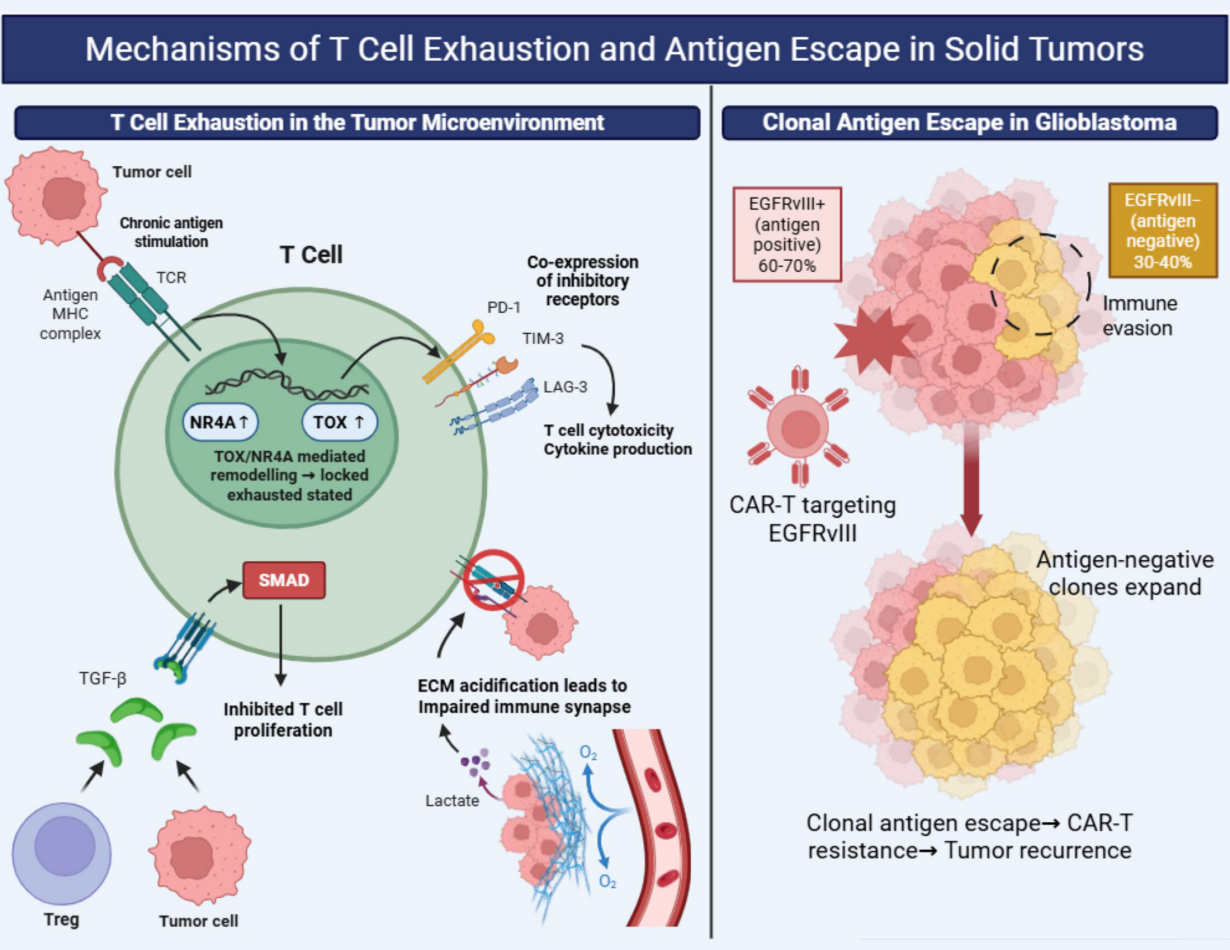
Mechanisms of T cell exhaustion and antigen escape in solid tumors. Left panel: Molecular mechanisms of T cell exhaustion in the TME. Sustained TCR signaling drives TOX upregulation, which promotes co-expression of inhibitory checkpoint receptors (PD-1, TIM-3, LAG-3). TGF-β/SMAD signaling further suppresses T cell proliferation. Lactic acid accumulation acidifies the ECM, impairing immune synapse formation. Epigenetic reprogramming by TOX and NR4A factors locks exhausted T cells in a hypofunctional state, addressable by CRISPR-mediated editing. Right panel: Clonal antigen escape in glioblastoma. Heterogeneous EGFRvIII expression across tumor clones results in selective elimination of antigen-positive cells following CAR-T infusion, with antigen-negative subclones expanding to drive resistance. ECM, extracellular matrix; LAG-3, lymphocyte-activation gene 3; MDSC, myeloid-derived suppressor cell; PD-1, programmed cell death protein 1; TIM-3, T cell immunoglobulin and mucin-domain containing-3; TOX, thymocyte selection-associated high mobility group box. References used (22, 23, 72, 99, 102–121).

Felsberg et al. presented a series of studies highlighting this issue in glioblastoma (GBM). Their work reported that epidermal growth factor receptor variant III (EGFRvIII) is tumor specific in approximately 40% of GBM cases (113). However, EGFRvIII mutations can arise during clonal evolution, resulting in EGFRvIII-mosaic tumors (113, 114). Furthermore, in an anti-EGFRvIII CAR-T trial in recurrent GBM, short-term safety and efficacy were observed, but durable responses failed because of adaptive resistance and antigen escape (115). O’Rourke et al. reported similar findings, showing that tumor cells from seven GBM patients exhibited a significant reduction in EGFR expression and increased expression of immunosuppressive markers (CD8, GRZMB, CD25, IDO1, PD-L1, and FoxP3) after a single infusion of EGFR-targeted CAR-T cells (23). These findings highlight how rapidly tumor heterogeneity and mosaicism can emerge. Luksik et al. similarly emphasized that unstable and heterogeneous antigen expression in GBM represents a major barrier to durable CAR-T efficacy, emphasizing the need for multi-target or adaptable antigen-targeting strategies (22). **Figure 3** summarizes the mechanisms of T Cell Exhaustion and antigen escape in solid tumors.

To address this challenge, several strategies are under investigation. Martins et al. proposed combining intratumoral CAR-T therapy with modulation of glioma-associated microglia and macrophages (GAM) using signal regulatory protein gamma–related protein (SGRP) to target both antigen-positive and bystander tumor cells, thereby improving clearance of mosaic GBM populations. Because the immune tumor microenvironment (iTME) in GBM limits immune checkpoint blockade efficacy, this approach may help mitigate immune resistance (122). In parallel, Arrieta et al. highlighted how tumor heterogeneity and immune escape mechanisms contribute to limited responses to immune checkpoint blockade in GBM, supporting the need for personalized and combination immunotherapy approaches (123).

Another strategy involves targeting multiple antigens using CAR-T. Dual-antigen targeting can enhance specificity and reduce antigen escape and may be achieved through combinations of distinct CAR-T products, bicistronic CAR-T cells, or tandem bispecific CAR-T constructs (116). Escobar et al. (2025) emphasized the potential of this approach in GBM, reporting that dual targeting with EGFR/IL-13Rα2 CAR-T and HER2/IL-13Rα2 CAR-T reduced antigenic heterogeneity while maintaining efficacy in preclinical models (117–119).

An additional approach involves adaptor molecule–dependent tumor killing, in which CAR-T cells are activated only in the presence of externally supplied adaptor molecules such as biotin, fluorescein isothiocyanate (FITC), or general control nondepressible-4 (GCN4). In this system, CAR-T cells eliminate tumor cells only when biotinylated, FITC-labeled, or GCN4-tagged tumor-targeting antibodies are administered (117, 124). Improved adaptor-based platforms, including universal CAR (UniCAR) and split, universal, and programmable (SUPRA) CAR systems, aim to reduce immunogenicity and improve safety. Two ongoing clinical trials (NCT04230265 and NCT04633148) are currently evaluating UniCAR, and preclinical studies have demonstrated reduced antigen escape and heterogeneity (117).

### 3.3 On-target, off-tumor toxicity and manufacturing barriers

A major safety concern in ACT is unintended damage to healthy tissues. Because many CAR-T targets are expressed at low levels in normal tissues, therapy may result in OTOT. In hematologic malignancies, examples of immune-related toxicities include CRS and ICANS (4, 125). Although these adverse events are generally manageable, toxicity becomes dose limiting in solid tumors.

Four clinical cases differentiate the severity and mechanism of dose-limiting OTOT. In CAIX-targeted CAR-T in ccRCC, although CAIX is expressed in >90% of ccRCC tumors, its presence on normal bile duct epithelium caused grade 2–4 hepatobiliary toxicity in four out of twelve patients (8). Those patients had significant liver enzyme elevations, with two patients requiring permanent treatment discontinuation, ultimately leading to premature trial termination (8). This case supported the claim that high tumor expression alone is insufficient to guarantee safety if even low-level expression exists in critical organs.

On the other hand, in HER2-directed CAR-T using high-affinity scFv, due to HER2 expression on normal lung tissue, fatal pulmonary toxicity occurred in the first patient to receive the highest dose (8). This event directly pushed the field toward affinity-directed CAR constructs that exploit the higher antigen density of tumor cells versus normal tissues. In CEA-targeted CAR-T in colorectal cancer, two patients developed severe watery diarrhea and colitis attributed to OTOT toxicity in CEA-expressing normal colonic mucosa (8), leading to development of hypoxia-responsive CEA-CARs designed to restrict T cell activation to the tumor’s hypoxic environment. In MAGE-A3 TCR-T study, fatal cardiogenic shock in two patients, attributed to molecular mimicry with a titin cardiac peptide, and fatal neurotoxicity in two of nine patients in a separate program due to off-target recognition in neural tissues, emphasize that antigen validation must include comprehensive cross-reactivity screening beyond the intended target (73–75).

Several strategies have been proposed to mitigate OTOT. Ai et al. (2024) suggested prioritizing tumor-selective developmental targets to enhance tumor-specific cytotoxicity (25). Claudin 6 (CLDN6) is highly expressed in germ cell tumors, epithelial ovarian cancer, and endometrial carcinoma, while remaining largely absent in normal tissues. In the phase 1/2 BNT211–01 trial, CLDN6-targeted CAR-T demonstrated meaningful efficacy in refractory solid tumors with manageable safety profiles, even at higher doses (24, 25). Another promising target is the receptor tyrosine kinase EphA2, which is overexpressed in solid tumors but minimally expressed in normal lung tissue (25).

Escobar et al. reported that incorporating inducible kill switches can improve safety by enabling selective elimination of CAR-T cells upon detection of toxicity (117). Examples include inducible caspase-9 (iCasp9) and herpes simplex virus thymidine kinase (HSV-TK), which can be activated using rimiducid or ganciclovir, respectively (117, 126, 127). Additional approaches use inactive receptor tags such as truncated EGFR, truncated HER2, or CD20 epitopes to enable targeted CAR-T depletion (117, 128–130). However, clinical results have been mixed. In one trial, iCasp9 effectively eliminated CAR-T cells, whereas in another study CAR-T cells re-expanded within six weeks and remained detectable for up to 30 months, potentially reflecting selective survival of cells expressing low levels of kill-switch components (13, 117). Other limitations include immunogenicity, interference with concurrent therapies, and reliance on an intact host immune system to clear CAR-T cells (117).

An alternative strategy involves SynNotch technology, or its fully humanized variant SNIPR, to regulate CAR expression in a tumor-specific manner (117, 131, 132). This system employs a synthetic notch receptor that recognizes a priming antigen with heterogeneous tumor expression, thereby inducing CAR-T activation only in tumor contexts. Although this approach reduces off-tumor activity, it does not completely eliminate risk.

Another major barrier to implementing CAR-T in solid tumors is manufacturing complexity and cost. Conventional autologous CAR-T production requires T cell collection, genetic modification, ex vivo expansion, and reinfusion (5, 133). This process is labor intensive, time consuming, and often requires prolonged clinical monitoring, with total treatment costs exceeding $350,000–$500,000 per patient in some settings (5, 133). Smolarska et al. further noted that autologous production results in extended manufacturing timelines and variable product quality, particularly in patients who have received prior cytotoxic therapies or have compromised immune function (1).

To address these limitations, allogeneic CAR-T approaches using healthy donor T cells have been proposed to enable standardized, off-the-shelf products (1, 133). However, this strategy carries a risk of graft-versus-host disease (GVHD). To mitigate this risk, gene-editing strategies such as β2-microglobulin knockout, TCR disruption, and HLA class I/II silencing have been implemented (1, 134, 135). Additional approaches use CRISPR-Cas9, TALEN, or zinc-finger nucleases (ZFNs) to eliminate endogenous TCRs, thereby reducing GVHD risk (133). Menegatti et al. demonstrated that deletion of Fas and B2M in CD3–Fas–CAR-T cells improved leukemia control in murine models by conferring resistance to immune-mediated rejection while preventing GVHD (136).

## 4 Managing toxicities of ACT in solid tumors

Toxicity remains a central limitation to the broader implementation of ACT in solid tumors. Although CAR-T, TCR-T, and TIL therapies have demonstrated meaningful antitumor activity, immune-mediated adverse events remain frequent and may be more difficult to predict and manage in solid tumor populations, where tumor burden, locoregional delivery strategies, and host factors differ from hematologic settings. Among these toxicities, cytokine release syndrome (CRS) and immune effector cell–associated neurotoxicity syndrome (ICANS) represent the most clinically significant and well-characterized complications.

### 4.1 Cytokine release syndrome

CRS is a potentially life-threatening systemic inflammatory response triggered by rapid immune activation following ACT. It is increasingly recognized in solid tumor settings, although incidence, severity, and kinetics vary depending on tumor burden, antigen distribution, cell product characteristics, and treatment protocol. The American Society of Clinical Oncology (ASCO) and the American Society for Transplantation and Cellular Therapy (ASTCT) define CRS as an uncontrolled immune response following immune effector cell infusion, characterized by fever at onset and potentially progressing to tachycardia, hypotension, hypoxia, headache, rash, myalgia, arthralgia, respiratory failure, coagulopathy, and multiorgan dysfunction (137, 138). Symptom onset typically occurs within 2–7 days after infusion but may be delayed for up to three weeks in some solid tumor trials (137–139).

CRS severity is graded according to ASTCT consensus criteria (138). Grade 1 CRS is defined by fever ≥38 °C without hypotension or hypoxia and is generally managed with supportive care, antipyretics, intravenous fluids, and symptomatic treatment, including β-blockers when indicated (140). Grade 2 CRS involves fever with hypotension not requiring vasopressors and/or hypoxia requiring low-flow oxygen. Patients require inpatient monitoring, and treatment commonly includes tocilizumab, an anti–IL-6 receptor monoclonal antibody, although rebound IL-6 signaling and potential neurotoxicity remain concerns (140). Grade 3 CRS is characterized by fever with hypotension requiring vasopressor support and/or hypoxia requiring high-flow oxygen. Management typically requires escalation to systemic corticosteroids, vasopressors, and consideration of alternative cytokine-targeting agents such as siltuximab (140). Grade 4 CRS represents life-threatening disease with refractory hypotension requiring multiple vasopressors and/or respiratory failure requiring mechanical ventilation. Management requires intensive care support and may include high-dose corticosteroids, IFN-γ blockade (emapalumab), JAK inhibition (ruxolitinib), or etoposide in the setting of immune effector cell–associated hemophagocytic syndrome (IEC-HS) (140).

Accumulating evidence suggests that early recognition and prompt intervention, particularly with tocilizumab and corticosteroids, can reduce progression to high-grade CRS without compromising antitumor efficacy (141). This has led to evolving clinical practice favoring earlier immunomodulatory intervention, especially in high-risk patients.

### 4.2 Immune effector cell–associated neurotoxicity syndrome

ICANS is a neurotoxic complication associated with immune effector cell therapies and reflects inflammatory injury to the central nervous system following immune activation (137). ASCO defines ICANS as a neurologic syndrome arising after activation of endogenous or infused immune effector cells (137). Clinical manifestations include encephalopathy, confusion, agitation, expressive aphasia, dysgraphia, tremor, myoclonus, fine motor impairment, seizures, and, in rare but severe cases, malignant cerebral edema (137, 142–144).

ICANS typically develops 3–9 days after infusion and may persist for 5–17 days, with aphasia among the most commonly reported early symptoms (143, 145). Diagnosis relies on structured neurologic assessment, including serial cognitive scoring, electroencephalography when seizures are suspected, and neuroimaging to exclude alternative etiologies. Cerebrospinal fluid analysis may be considered in refractory or atypical cases (137).

Management strategies depend on severity. Low-grade ICANS is generally treated with supportive care and close neurologic monitoring. Corticosteroids remain first-line therapy for moderate to severe ICANS. For steroid-refractory cases, IL-1 blockade with anakinra is increasingly employed, although prospective randomized evidence remains limited (146, 147). Other investigational approaches include lenzilumab and defibrotide, aimed at mitigating endothelial and cytokine-mediated injury (137). Most low-grade cases resolve spontaneously, but severe ICANS requires urgent intervention because of the risk of long-term neurologic morbidity or mortality (137, 143, 145).

### 4.3 Other toxicities, risk stratification, and prevention

Beyond CRS and ICANS, ACT in solid tumors is associated with a spectrum of additional clinically relevant toxicities. These include prolonged cytopenias, infectious complications, B cell aplasia, movement disorders, hemophagocytic lymphohistiocytosis–like syndromes, and organ-specific immune-mediated adverse events such as myocarditis, colitis, hepatitis, and dermatitis (143, 144, 147). Movement disorders have emerged as a delayed complication, particularly following anti–BCMA CAR-T therapy, and may present weeks to months after infusion, although underlying mechanisms remain incompletely understood (144). TIL therapy and high-dose IL-2 regimens carry additional toxicity risks, including capillary leak syndrome, severe hypotension, renal dysfunction, and pulmonary edema, requiring specialized supportive care and intensive monitoring (143).

A hypothesized structured pre-infusion risk stratification should include three categories of predictors:

At the patient level, age ≥65 years, ECOG performance status ≥2, significant cardiopulmonary comorbidity, and an elevated baseline inflammatory state (ferritin >500 ng/mL, CRP >10 mg/L, or LDH elevation) identify patients at high risk for severe CRS and prolonged toxicity (144, 147). At the disease level, high tumor burden, high antigen density at the target site, and extensive prior cytotoxic therapy, each act as independent risk factors. In the setting of solid tumors, locoregional delivery routes modify but do not eliminate the risk of toxicity. For example, intraventricular delivery concentrates immune activation at the CNS compartment, producing site-specific neurotoxicity, while intrapleural delivery may cause pleuritis, both of which require distinct site-specific surveillance protocols (14, 15, 21). Moreover, multiple product-level factors also affect toxicity risk, including the CAR-T costimulatory domain, T cell phenotype at infusion, cell dose, and manufacturing modality. CD28-containing constructs tend to produce faster and more intense CRS compared to 4-1BB, and a high proportion of effector memory T cells at infusion correlates with greater risk of CRS (138, 140).Post-infusion surveillance should be done based on defined trigger thresholds: fever ≥38 °C within 72 hours, ferritin doubling within 24 hours, or CRP exceeding 10 mg/L, each representing an early warning sign for grade ≥2 CRS requiring immediate consideration of tocilizumab (144–147). Given that solid tumor CRS kinetics may be slower and more attenuated than in hematologic malignancies, surveillance intervals should extend to at least 14 days post-infusion (144–147).

Prevention and mitigation strategies increasingly emphasize proactive or early intervention with tocilizumab, anakinra, or low-dose corticosteroids, along with coordinated multidisciplinary management involving oncology, critical care, neurology, and infectious disease specialists. In parallel, advances in cellular engineering aim to improve safety through the development of lower-affinity CAR constructs, reversible or inducible safety switches, and controllable activation platforms that reduce the risk of severe immune-mediated toxicity (144–146). Systematic adverse event reporting, real-world safety registries, and continued translational research remain essential to refining toxicity prediction, optimizing risk stratification models, and improving the overall safety profile of ACT in solid tumors (142, 143).

## 5 Future clinical directions and strategies to overcome challenges

The translation of ACT success from hematological malignancies to solid tumors represents the current frontier in immuno-oncology. While first-generation CAR-T cells have achieved curative status for diverse B cell leukemias and lymphomas, their efficacy in solid tumors remains sporadic (148). This discrepancy arises from profound differences in the TME. Unlike fluid hematological compartments, solid tumors construct complex physical and immunological fortresses that actively exclude, suppress and exhaust infiltrating T cells (148). Consequently, future clinical directions of ACT are pivoting away from mere target discovery toward holistic engineering strategies designed to overcome these multi-layered barriers. Next-generation clinical approaches rely on three integrated pillars: sophisticated genetic “armoring” and logic-gating of therapeutic cells; rational combination strategies that remodel the TME; and advanced delivery and patient selection modalities to ensure these living drugs reach the right target and patient.

### 5.1 Multi-antigen targeting strategies

The primary mechanism of late treatment failure in CAR-T and TCR-T approaches have been attributed to antigen heterogeneity and clonal escape (117–119). Hence, tandem bispecific CAR constructs are designed to simultaneously present two scFv domains within a single CAR molecule, enabling parallel recognition of two distinct antigens without requiring two separate cell products (117–119). Preclinical GBM models using EGFR/IL-13Rα2 and HER2/IL-13Rα2 tandem CARs demonstrated that bivalent targeting reduced antigen escape while maintaining robust cytotoxicity (117–119). Moreover, bicistronic vectors encoding two CARs from a single locus, and combinations of two discrete CAR-T products, offer complementary approaches. Adaptor-based universal platforms, such as UniCAR and SUPRA-CAR, further extend this concept by enabling programmable, switchable multi-antigen coverage without requiring tumor-specific manufacturing for each antigen combination (117, 124).

Hence, tandem bispecific CAR constructs represent the most pragmatic short-term solution to antigen heterogeneity, as they can be incorporated into existing autologous manufacturing workflows without fundamental redesign. Whether the coverage of two antigens is sufficient given the degree of heterogeneity seen across most solid tumors, or whether adaptive antigen loss will eventually emerge regardless, remains an open question. To resolve this, we advocate for mandatory quantitative antigen escape monitoring as a correlative endpoint in all future multi-antigen CAR-T trials in solid tumors.

### 5.2 Armored and logic-gated CAR designs

The most immediate solution to T cell dysfunction in the TME lies in intrinsic modification of the therapeutic product (16, 149). First-generation CAR-T cells proved highly vulnerable to immunosuppressive soluble factors and checkpoint ligands ubiquitously present in solid tumors. To counter this vulnerability, “armored” CAR-T cells are now being engineered not only to resist suppression but to actively remodel the microenvironment (16, 149). A landmark advancement includes dual-function CAR–T cells engineered to secrete fusion proteins that simultaneously stimulate immunity and block checkpoint axes (16, 149). For example, in a preclinical study, CAR-T cells engineered to secrete an anti-PD-L1–IL-12 fusion protein eradicated advanced prostate and ovarian tumors in murine models, while avoiding the systemic toxicity historically associated with IL-12. This “localized attack” strategy marks a critical shift toward safer, potent multimodal cell therapies (149). As a proof-of-concept for CAR-T armoring with specific TME resistance mechanisms, the CART-PSMA-TGFβRDN phase I trial delivered exactly that (16). PSMA-targeting CAR-T cells that were engineered with a dominant-negative TGF-β receptor demonstrated CAR-T expansion, with evidence of PSA reductions of ≥30% in 4/13 patients, and hence providing evidence that TGF-β blockade can counteract one of the principal immunosuppressive mechanisms of the prostate tumor TME (16). This trial acted as the first human validation of this armoring strategy (16).

Addressing on-target, off-tumor toxicity becomes increasingly critical as ACT moves toward antigens expressed at low levels on healthy tissue. The field now advances “logic-gated” T cells that require multiple signals to activate cytotoxicity. The EVEREST 2 trial (NCT06051695) exemplifies this innovation: it is a first-in-human, phase 1/2 study evaluating A2B694, an autologous logic-gated Tmod™ CAR-T therapy designed to target mesothelin while using a blocker receptor recognizing HLA-A*02 to distinguish between malignant and normal cells—an “AND-NOT” logic gate that addresses OTOT toxicity (27, 28). Early results from the dose-escalation phase reveal manageable safety with no dose-limiting toxicities, CRS or neurotoxicity, and demonstrate CAR-T expansion, persistence and tumor infiltration including in a pancreatic-cancer biopsy 42 days post-infusion (27, 28). This study remarkably achieved a complete response in a patient with refractory metastatic non-small-cell lung cancer, hence acting as a landmark for precision ACT in solid tumors (27, 28). SynNotch and SNIPR systems are also able to provide an alternative logic-gating mechanism that requires sequential recognition of a priming antigen before CAR transcription is initiated (117, 131, 132).

In summary, logic-gating represents a major paradigm shift, because it splits antigen expression from toxicity risk, which is a fundamental constraint that limits antigen selection in solid tumors. Similarly, the CART-PSMA-TGFβRDN trial data (16) establishes TGF-β armoring as a validated strategy worthy of broader expansion. Logic-gated platforms may be most impactful in tumor types where a candidate antigen has demonstrated efficacy but is historically limited by OTOT concerns, such as in MSLN-expressing PDAC, pleural mesothelioma, and ovarian cancer.

### 5.3 Metabolic and cytokine engineering

The metabolic competition between tumors and infiltrating T cells represents an underappreciated but actionable barrier in solid tumor ACT (99, 102). Within the TME, aerobic glucose consumption and lactate accumulation suppresses immune synapse formation and decreases T cell motility and function (99, 102). Engineering metabolic resilience into CAR-T and TCR-T cells is therefore a priority for solid tumor settings to overcome the TME barrier. Current strategies include overexpression of metabolic enzymes such as argininosuccinate synthetase that compensates for Arg-1–mediated arginine depletion that is released by MDSCs. Other strategies include the introduction of transgenic IL-15 or IL-21 autocrine signaling loops to reduce dependence on exogenous cytokines for persistence, and CRISPR-mediated PD-1 knockout to prevent checkpoint-driven metabolic exhaustion (120, 121).

Other promising engineering targets are the transcriptional and epigenetic regulators of T cell exhaustion, such as TOX-driven chromatin remodeling, NR4A factors, and mitochondrial biogenesis programs, all of which can reprogram CAR-T cell fate toward long-lived effector memory rather than terminal exhaustion (72, 112). In addition to that, CRISPR-engineered PD-1 knockout T cells have also shown enhanced persistence and antitumor activity in early clinical and preclinical settings (120, 121), potentially removing the need for simultaneous systemic anti-PD-1 antibody therapy.

Metabolic engineering remains the least clinically advanced of the five strategies mentioned, yet we regard it as essential for durable responses in metabolically hostile solid tumor TMEs. In our view, autocrine IL-15 or IL-21 signaling offers the most direct mechanism to improve persistence without increasing systemic cytokine toxicity and should be incorporated into next-generation armored CAR constructs as a standard design element. PD-1 knockout represents a cleaner solution than concurrent anti-PD-1 antibody therapy and should be advanced as a standalone engineering modification in Phase I trials across multiple solid tumor types. Lastly, epigenetic reprogramming of CAR-T cell exhaustion through TOX or NR4A targeting is the most compelling emerging frontier in this field.

### 5.4 Allogeneic and gene-edited ACT products

Conventional autologous CAR-T production usually requires 3–4 weeks of manufacturing per patient at costs exceeding $350,000–$500,000, posing a logistical challenge especially with the rapid disease progression seen in most advanced solid tumors (1, 5, 133). Allogeneic “off-the-shelf” CAR-T cells from healthy donors offer a ready-made product scalable to population-level use (1, 5, 133). CRISPR/Cas9 is the current gold-standard tool for knocking out endogenous TCR to prevent graft versus host disease (GVHD) and HLA class I molecules to reduce probability of host rejection in donor T cells (134, 135). Early-phase trials suggest that CRISPR-edited allogeneic CAR-T cells can persist and function in solid tumor settings, however, its persistence duration remains shorter than autologous products in immunocompetent hosts (134, 135). Recent preclinical models such as Menegatti et al. demonstrated that combined Fas and B2M deletion in CD3– allogeneic CAR-T cells had resistance to immune-mediated rejection in murine leukemia models while also preventing GVHD (136). Moreover, TALEN and zinc-finger nuclease approaches were also able to provide alternative precise editing strategies beyond CRISPR.

Allogeneic platforms will be essential for broadening access to ACT. However, immunological persistence in solid tumor patients remains the main unresolved challenge. All allogeneic solid tumor ACT trials should mandate immune profiling as primary endpoints, since without mechanistic data on rejection kinetics and residual host immune competence, it becomes impossible to distinguish true platform failure from insufficient lymphodepletion. Advancing allogeneic products in solid tumors without parallel development of more intensive conditioning regimens or additional immune evasion strategies, such as HLA-E expression to evade NK cell-mediated killing, would be premature.

### 5.5 Locoregional delivery approaches

The physical route of administration is another factor that deserves re-evaluation. Intravenous infusion frequently results in first-pass lung entrapment of T cells with poor subsequent trafficking to solid tumor sites (150). Locoregional delivery methods, such as intrapleural injection for mesothelioma or intratumoral injection for accessible masses, have emerged as powerful options to maximize effector-cell concentration at the disease site while minimizing systemic exposure (150). These local interventions can also synergize with systemic immunity by inducing immunogenic cell death, effectively turning the tumor into an *in-situ* vaccine (150).

The intrapleural approach has the most robust clinical evidence base among locoregional approaches (26). In a first human phase trial, Adusumilli et al. conducted intrapleural mesothelin-targeted CAR-T cells (0.3M–60M cells/kg) in 27 patients with malignant pleural disease, predominantly mesothelioma, demonstrating that the approach was safe and well tolerated, with CAR-T cells detectable in peripheral blood beyond 100 days in 39% of patients (26). Among 18 patients who subsequently received pembrolizumab, the median overall survival from CAR-T infusion was 23.9 months, with a one-year overall survival was 83%, and stable disease was sustained for ≥6 months in 8 patients, while 2 patients exhibited complete metabolic responses on PET scan, with no high-grade CRS or ICANS observed (26). This trial acted as the clinical and mechanistic proof-of-concept of intrapleural CAR-T delivery. By concentrating effector cells within the tumor compartment and activating systemic immunity through tumor antigen release, synergizes with subsequent checkpoint blockade to convert immunologically “cold” tumors into responsive ones (26).

Complementary evidence was provided in GBM studies. Intracerebroventricular bivalent EGFR/IL-13Rα2 CAR-T achieved 62% tumor regression in patients with no grade 4–5 neurotoxicity at the MTD, establishing both feasibility and bioactivity of intraventricular delivery (15). Similarly, intraperitoneal delivery for ovarian cancer and PDAC achieved high local concentrations in anatomically appropriate disease compartments (18, 150). Intratumoral injection for accessible solid masses has shown synergy with systemic checkpoint inhibition by inducing immunogenic tumor cell death, effectively converting a treated lesion into an *in-situ* vaccine and generating systemic immune responses (18).

In our view, intrapleural and intraventricular delivery routes have accumulated sufficient feasibility and efficacy data to justify progression into randomized comparisons with systemic administration within the next five years, specifically in mesothelioma and GBM, respectively. The field should not continue generating single-arm feasibility data in these indications without a comparative arm. Randomization to intravenous versus locoregional delivery, with tumor infiltration kinetics as a co-primary endpoint alongside response rate, would definitively establish the clinical value of route optimization.

### 5.6 Biomarkers and patient selection for ACT in solid tumors

Patient selection for ACT requires moving beyond simple antigen expression profiling toward integrated predictive biomarkers. Based on current evidence, available biomarkers can be broadly categorized into those approaching clinical implementation and those still under active investigation.

Among biomarkers approaching clinical implementation, antigen density quantification has been used as a prospective enrollment criterion in the satri-cel gastric cancer trial, where quantitative IHC-based antigen scoring (e.g., CLDN18.2 H-score ≥75) correlated with improved ORR (12). Antigen density measured by flow cytometry on circulating tumor cells offers a less invasive complement to biopsy-based IHC. Another biomarker is a high baseline intratumoral CD8+ TIL density and a favorable CD8:Treg ratio that were able to independently predict TIL product quality, post-infusion expansion, and clinical response across melanoma, NSCLC, and cervical cancer TIL trials (57–59, 61). Both metrics are increasingly incorporated as co-primary endpoints in ongoing and upcoming trials. Pre-infusion inflammatory biomarkers, such as ferritin >500 ng/mL and CRP >10 mg/L at the time of CAR-T infusion were found to be associated with grade ≥2 CRS in both hematologic and solid tumor ACT cohorts and hence should be integrated into risk stratification algorithms for clinical decision-making regarding prophylactic intervention (144, 147).

Several biomarkers remain under active investigation. Higher TCR clonality within the infused TIL product and in circulating T cells at day 14 post-infusion correlates with improved PFS in melanoma TIL trials (61). However, prospective validation in randomized trials is required before use in patient selection. Tumor mutational burden (TMB) has been hypothesized to increase neoantigen density, potentially improving TIL and TCR-T reactivity. However, TMB thresholds validated for checkpoint blockade may not translate directly to ACT, and prospective data in ACT-specific cohorts remain limited (151). Moreover, TGF-β–high TME gene expression signatures predict poor TIL engraftment, and co-expressed exhaustion gene sets (TOX, PD-1, TIM-3, LAG-3) can identify patients that are most likely to require armored or checkpoint-resistant cell products (72, 111, 112). In addition to that, early ctDNA clearance post-infusion (e.g., at day 14 or 28) is being evaluated as a dynamic response biomarker in several ongoing ACT trials, with prospective validation still pending (151). Lastly, emerging evidence suggests that TP53-mutant tumors may show differential resistance to standard CAR-T constructs, though this remains hypothesis-generating, and including TP53 status in the standard molecular characterization panel of future ACT trials in solid tumors would be a reasonable next step (151).

The most critical unmet translational need in the ACT field is not the discovery of new biomarkers but the prospective validation of existing candidates in randomized trial designs. Mandatory pre-specified biomarker analysis plans in all Phase II and III solid tumor ACT trials, with antigen density and baseline TIL density as co-primary endpoints are needed. Without this investment, the field will continue producing efficacy data without the mechanistic foundation needed to improve patient selection and treatment adaptation.

In summary, the future of ACT in solid tumors is shifting from incremental improvements of first-generation products to a comprehensive ecosystem of innovations: engineered cellular products, rational combination therapies, novel delivery routes, and precision patient matching. Overcoming the formidable barriers posed by solid tumor TME will require this multifaceted approach, and early clinical and pre-clinical data give cause for cautious optimism.

## 6 Conclusions

Adoptive cell therapy, including CAR-T cell therapy, TIL, and TCR-T approaches represents a promising frontier for the treatment of solid tumors. However, multiple biological and practical barriers continue to limit its broad clinical success. Tumor antigen heterogeneity remains a central challenge, driving antigen escape and treatment resistance, while on-target, off-tumor toxicity raises safety concerns when target antigens are shared with healthy tissues. In addition, the logistical complexity, cost, and variability of autologous CAR-T manufacturing constrain scalability and equitable access. Ongoing advances in multi-antigen targeting, adapter-based CAR platforms, safety-switch systems, SynNotch-based regulation, and allogeneic manufacturing strategies offer viable pathways to overcoming these limitations. Emerging clinical evidence, across melanoma, gastric cancer, malignant pleural mesothelioma, neuroblastoma, and sarcoma, demonstrates that innovative CAR-T designs can improve tumor coverage, mitigate toxicity, and enhance durability of response. Future progress will depend on integrating refined antigen selection, tumor microenvironment modulation, scalable manufacturing platforms, and rigorous clinical validation. Continued translational research and thoughtfully designed clinical trials will be essential to realize the full therapeutic potential of adoptive cell therapy in solid tumors.
